# Effect of Raw and Fermented Grape Pomace on the Growth Performance, Antioxidant Status, Intestinal Morphology, and Selected Bacterial Species in Broiler Chicks

**DOI:** 10.3390/ani11020364

**Published:** 2021-02-01

**Authors:** Emrah Gungor, Aydin Altop, Guray Erener

**Affiliations:** Department of Animal Science, Faculty of Agriculture, Ondokuz Mayis University, 55200 Samsun, Turkey; aaltop@omu.edu.tr (A.A.); gerener@omu.edu.tr (G.E.)

**Keywords:** grape by-product, grape pomace, growth performance, antioxidant status, chick

## Abstract

**Simple Summary:**

Grape pomace (GP) is a by-product of fruit juice and wine production. Owing to its biochemical composition and lower cost, GP has the potential to be a feed additive for poultry nutrition. However, its antinutritional compounds limit its usability in broiler diets. Fermentation is an ancient and useful method for the utilization of agricultural residues. The aim of the study was to investigate the effects of GP and *Aspergillus niger*-fermented grape pomace (FGP) on the growth performance, antioxidant capacity, intestinal morphology, and selected bacterial species in broilers. Dietary GP improved the antioxidant status and intestinal morphology of broiler chickens. Dietary FGP enhanced the growth performance, antioxidant capacity, and selected intestinal bacterial species of broilers. Dietary GP caused worsened growth performance compared with the synthetic antioxidants, although FGP had similar growth performance. The findings demonstrate that FGP can be an alternative to synthetic antioxidants for broiler diets.

**Abstract:**

The effects of raw (GP) and fermented grape pomace (FGP) on the growth performance, some carcass parameters, antioxidant status, intestinal morphology, and selected bacterial species in broiler chicken were investigated in this study. Grape pomace was fermented with *Aspergillus niger* for 7 d. In total, 140 one-day-old male chicks (Ross 308) were randomly assigned to four treatment groups, with five replicates and seven birds each. Chickens were fed either a basal diet (CON) or the basal diet supplemented with 0.25 g/kg synthetic antioxidants (5% butylated hydroxytoluene, 1% butylated hydroxyanisole, and 11% ethoxyquin) (AO), or 15 g/kg GP (GP), or 15 g/kg FGP (FGP) for 42 d. Dietary GP raised serum glutathione peroxidase (*p* = 0.031) and superoxide dismutase (*p* = 0.021) levels, increased ileum lamina muscularis thickness (*p* = 0.016), and did not affect selected bacterial species in the cecum of broiler chickens. Dietary FGP improved body weight (*p* = 0.003), increased the serum catalase level (*p* = 0.032), and decreased the cecal *Clostridium perfringens* count (*p* = 0.033) but did not affect the ileal morphology of broiler chickens. The carcass parameters, malondialdehyde level, pH, and color of the breast meat of chickens were not changed by either GP or FGP supplementation. Chickens fed with the synthetic antioxidants had similar growth performance with the chickens fed with FGP but had better body weight (*p* = 0.003) and feed conversion ratio (*p* = 0.045) compared with the chickens fed with GP. The obtained results showed that FGP can be used as an alternative to synthetic antioxidants in broiler diets.

## 1. Introduction

Oxidative stress is one of the most important factors affecting the growth performance of broilers and profitability in intensive poultry production [1]. It can also increase mortality by reducing disease resistance and can impair meat quality such as color, odor, texture, and nutritional composition [2]. The use of synthetic antioxidants such as butylated hydroxytoluene (BHT), butylated hydroxyanisole (BHA), and ethoxyquin (EQ) has been a common practice in poultry nutrition to avoid the negative effects of oxidative stress on chickens [3]. However, consumers are increasingly concerned about synthetic antioxidants in recent years as synthetic antioxidants have toxic and carcinogenic effects [4] and are also retained in broiler meat [5]. Therefore, researchers have focused on potential natural antioxidants that can be used in broiler diets as substitutes for synthetic antioxidants.

Grapes (*Vitis vinifera* L.) belonging to the Vitaceae family are the most produced fruit in the world, with annual production exceeding 79 million tons [6]. Grape pomace (GP) is a by-product of the fruit juice and wine industry, consisting of skin, seeds, and stems [7]. It is produced as 20% of the total weight of grapes processed in the factory [8]. It contains a large quantity of phenolic compounds such as (+)-catechins, (−)-epicatechin and (−)-epicatechin-3-O-gallate, and procyanidins [9]. Therefore, GP has a strong antioxidant effect [10] and antibacterial effect against various pathogenic bacteria such as *Escherichia coli* and *Staphylococcus aureus* [11]. Grape pomace also has growth-stimulating [12], immune-enhancing [13], and antilipidemic [14] effects on broiler chickens.

In recent years, solid-state fermentation has been a useful method for utilizing agricultural by-products in poultry diets, which refers to microbial growth in moistened solid media. Fermented feeds have positive effects on the growth performance, gut microbiota, and morphology of broiler chickens [15]. *Aspergillus niger* is preferred in solid-state fermentation due to the ability to grow successfully in a low-water environment [16]. *Aspergillus niger* can produce digestive enzymes such as amylase, protease, cellulase, xylanase, lipase, and tannase [17], and is also used as a probiotic fungus in poultry nutrition [18]. It can eliminate the antinutritional compounds in agricultural by-products [19]. It can also increase the antioxidant and antimicrobial activity of the substrate by raising the amount and/or effectiveness of polyphenolic compounds [20]. *Aspergillus niger* can be thought to make a more functional product from GP by solid-state fermentation considering all these features. This study aimed to investigate the effects of GP and fermented grape pomace (FGP) on growth performance, antioxidant status, intestinal morphology, selected bacterial species, some carcass characteristics, and meat and liver quality in broiler chickens.

## 2. Materials and Methods

All experimental procedures were performed strictly in accordance with guidelines and were approved by the local Ethics Committee of Ondokuz Mayis University (protocol number: 2018/10).

### 2.1. Preparation of FGP and Chemical Analysis

Grape pomace was obtained from a fruit juice factory and was quickly sun-dried and milled to a size of 2 mm and stored at 4 °C until the fermentation process. It was divided into two groups. One group was untreated (unfermented; raw) and the other group was fermented using *A. niger* (ATCC 9142). Grape pomace was enriched with a nutrient solution (glucose, urea, (NH_4_)_2_SO_4_, peptone, KH_2_PO_4_, MgSO_4_, and distilled water: 40, 20, 60, 10, 40, 1 g, and 1 L, respectively, for each kg of grape pomace) and sterilized with an autoclave at 121 °C for 15 min in fermentation bags to remove native yeast and bacteria. *Aspergillus niger* was inoculated in the fermentation medium by estimating 10^5^ spores per kg of grape pomace. All microbiological procedures were performed in a sterile cabin to prevent possible contamination. The GP was incubated at 30 °C for 7 d with gentle mixing once a day. After fermentation, the FGP was dried on polyethylene sheets at room temperature to reach 90% dry matter. The FGP was again ground to a size of 2 mm and given to birds without storage. The GP and FGP were analyzed for crude protein (method, 976.06), ether extract (method, 920.29), ash (method, 942.05), and crude fiber (method, 973.18) according to AOAC [21]. Condensed tannin analysis was performed by the method described by Makkar et al. [22]. Radical scavenging activity was determined with the DPPH method as described by Brand-Williams et al. [23].

### 2.2. Animals and Diets

A total of 140 one-day-old male broiler (Ross 308) chicks were allocated to 4 dietary treatments in a completely randomized design. Each treatment included 5 replicates of 7 birds. The dietary treatments were (i) a basal diet (CON), (ii) the basal diet supplemented with 15 g/kg GP (GP), (iii) the basal diet supplemented with 15 g/kg FGP (FGP), and (iv) the basal diet supplemented with 0.25 g/kg synthetic antioxidants (AO; 5% BHT, 1% BHA, and 11% EQ). The dosage of the synthetic antioxidants was chosen according to the recommendation of the producer company. The basal diet was formulated to meet the requirements of broilers according to the recommendations of the Ross breeders for Ross 308 male birds (Table 1). The birds were housed in floor pens (100 × 115 × 70 cm^3^) and had free access to mash feed and fresh water. The initial temperature of 32 °C was kept for 3 d and was gradually reduced until it reached 20 °C at 42 d of age. The vaccination program was designed as an infectious bursal disease vaccine on day 20 and a Newcastle disease vaccine on day 26.

### 2.3. Performance, Carcass Characteristics, Meat and Liver Quality

Body weight (BW) and feed intake (FI) were measured weekly for each replicate. The feed conversion ratio (FCR) was calculated and adjusted by daily recorded mortality. At 42 d of age, one bird from each replicate representing the mean weight of the pen was selected, slaughtered, and eviscerated. The visceral organs (heart, liver, gizzard, and spleen), abdominal fat, and gastrointestinal tract were weighed and expressed as a percentage of live body weight. The dressing percentage was calculated as hot carcass weight as a percentage of live weight. The breast muscle (right side) and liver were packed individually in labeled plastic bags and stored at 4 °C for measurement of color and pH values at 1, 5, and 11 d of storage. The color indices (L*: lightness, a*: redness and b*: yellowness) were measured from three locations of each sample by a Chroma Meter (CR300, Konica Minolta, Osaka, Japan). The pH values of breast meat and liver were determined by using a digital pH meter (Testo 205, Testo AG, Lenzkirch, Germany).

### 2.4. Serum GPx, SOD, and CAT Levels

The blood samples were collected with a syringe from the branchial vein of 5 birds from each treatment (one bird per replicate). The samples were immediately transferred to serum separator tubes preventing hemolysis and centrifuged at 3000 g at 4 °C for 10 min. The serum was separated and stored at −20 °C until further analysis for glutathione peroxidase (GPx), superoxide dismutase (SOD), and catalase (CAT). The serum biochemical measurements were determined by commercial ELISA kits (GPx, CK-bio-20413; SOD, CK-bio-19400; CAT, CK-bio-18096; Shanghai Coon Koon Biotech, Shanghai, China) using an ELISA Plate Reader (RT-2100C, Rayto, Shenzhen, China). The manufacturer’s instructions were followed for each analysis.

### 2.5. Meat MDA Level

The right-side breast meat samples were used to determine the malondialdehyde (MDA) level at different storage periods of 45 min and 11 d at 4 °C. The MDA concentration of breast meat was measured by determining the thiobarbituric acid-reactive substances (TBARS) according to the procedure described by Tarladgis et al. [24] using a spectrophotometer (GENESYS 10S UV–Vis, Thermo Fisher Scientific, Waltham, MA, USA). The concentration of MDA in breast meat samples is given as milligrams per kilogram of wet meat.

### 2.6. Histologic Analysis of the Ileum

At slaughter, ileum samples were collected from one bird in each replicate for histological analysis. The samples were cut into 1.5 cm pieces and fixed in 10% formalin, processed in paraffin, and cut to 5 µm sections with a microtome (Leica, Nussloch, Germany). Sections were stained with hematoxylin and eosin methods and examined with an optical microscope (Primo Star, Zeiss, Jena, Germany) to determine the villus height (VH), crypt depth (CD), and lamina muscularis mucosa thickness (LMT).

### 2.7. Enumeration of Bacteria Population in the Cecum

Cecal samples were collected aseptically during evisceration and stored at −20 °C until the enumeration of the bacteria population. Ten-fold serial dilutions of each sample were prepared in sterile Ringer solution from 10^−1^ to 10^−9^. Then, 100 µL of the different dilutions were separately dropped and spread onto selective agar plates. *Lactobacillus* spp. was enumerated on de Man, Rogosa, and Sharpe agar (Merck 110660) after anaerobic incubation at 30 °C for 72 h. *Enterococcus* spp. was enumerated on Slanetz and Bartley agar (Merck 105262) at 37 °C for 48 h. *E. coli* was enumerated on Eosin Methylene Blue agar (Merck 101347) at 35 °C for 24 h. *Campylobacter jejuni* was enumerated on *Campylobacter* selective agar after microaerophilic incubation at 42 °C for 48 h. *S. aureus* was enumerated on Baird-Parker agar (Merck 105406) after incubation at 37 °C for 48 h. *Clostridium perfringens* was enumerated on Tryptose Sulfite Cycloserine agar (Merck 111972) with anaerobic incubation at 37 °C for 24 h. The microbial colonies were counted in three replicated plates and the average was taken. The total population was expressed as log_10_ colony-forming units (CFU) per gram of wet cecal content.

### 2.8. Statistical Analysis

All data were analyzed in a completely randomized design with one-way ANOVA using SPSS software (Version 21.0, SPSS, Chicago, IL, USA). Significant differences between treatments were determined by Duncan’s multiple range tests. All results are presented as the mean and pooled standard error of the mean (SEM). Each pen was used as the experimental unit for growth performance; an individual bird served as the experimental unit for carcass characteristics, meat quality, antioxidant capacity, intestinal morphology, and selected bacterial species. A Chi-square test was performed to analyze the mortality rates. Microbial counts were log-transformed before analysis. The level of statistical significance was set at *p* < 0.05.

## 3. Results

### 3.1. Solid-State Fermentation

The nutritional composition and radical scavenging activity of GP and FGP are presented in Table 2. The fermentation process increased (*p* < 0.001) the crude protein, ash, and crude fiber content of GP. Ether extract (*p* = 0.029), nitrogen-free extract (*p* < 0.001), and radical scavenging activity (*p* = 0.006) were decreased after solid-state fermentation. However, condensed tannin was not affected by solid-state fermentation.

### 3.2. Growth Performance

The mortality rate of broilers was 2.86% and did not differ among the dietary treatments.

The chicks fed with FGP and AO had higher BW (*p* = 0.003) compared with the birds in the CON group (Table 3). The birds from the AO group had higher BW (*p* = 0.003) and lower FCR (*p* = 0.045) compared with that of chicks from the GP group.

### 3.3. Carcass Characteristics

No statistical difference was observed in the dressing percentage and relative weights of heart, liver, gizzard, gastrointestinal tract, abdominal fat, spleen, and edible internal organs between treatment groups (data not shown).

### 3.4. Meat and Liver Quality

Dietary treatments had no effect on the pH and L*, a*, and b* values of breast meat at 1, 5, and 11 d of storage (data not shown). However, the L* value of the liver was increased in the chicks from the AO group compared with the birds in the CON group at 1 (*p* = 0.032) and 11 d (*p* = 0.034) of storage (Table 4). The chicks in the AO group had lower pH value (*p* = 0.030) in liver than the broilers from the other groups. Liver of the chicks in the AO group also had the highest b* value (*p* = 0.017) among the treatment groups.

### 3.5. Antioxidant Status

The serum GPx level of the chicks fed GP was higher (*p* = 0.031) than that of the birds from the CON and AO groups, but not statistically different from that of the chicks in the FGP group (Table 5). Similarly, the chicks fed GP had the highest serum SOD level (*p* = 0.021) among the treatment groups. However, the serum CAT level of the broilers in the FGP group was higher (*p* = 0.032) than that of the chicks from the other treatment groups. Dietary treatments did not change the MDA level of breast meat at 45 min and 11 d of storage (data not shown).

### 3.6. Intestinal Bacterial Species

The cecal *C. perfringens* count was lower (*p* = 0.033) in the chicks fed FGP compared with the other treatment groups (Table 6). Dietary treatments did not affect the cecal *Lactobacillus* spp., *Enterococcus* spp., *E. coli*, *C. jejuni,* and *S. aureus* counts in broilers.

### 3.7. Intestinal Morphology

The VH (*p* < 0.001) and villus height to crypt depth ratio (VH:CD; *p* = 0.041) of the chicks fed AO were higher than those of the birds in the CON groups (Table 7). Chicks from the GP group had higher LMT (*p* = 0.016) compared with the birds in the FGP group. The CD was not affected by dietary treatments.

## 4. Discussion

### 4.1. Solid-State Fermentation

Solid-state fermentation can improve the nutritional composition of agricultural residues [25]. Similar to the result of the study, *A. niger* increased the crude protein content of grape seed in solid-state conditions [26]. Similarly, Dhillon et al. [27] reported an increase in protein content of GP after *A. niger* solid-state fermentation. Increase in the crude protein may be attributed to produced enzymes and/or mycelia by *A. niger* [28]. Mycelia of *A. niger* may also be the reason for the increase in the crude fiber content because its cell wall is rich in cellulose [29]. Similar to the results of the study, Altop et al. [26] showed that *A. niger* decreased the ether extract content of grape seed.

Soluble carbohydrates are preferred over other nutrients by *A. niger* to use as a carbon source [30]. A decrease in the nitrogen-free extract can be attributed to its possible consumption by *A. niger*. Similarly, Altop et al. [26] reported a decrease in the nitrogen-free extract of grape seed with *A. niger* fermentation.

Increase in ash content was considered as a relative increase rather than an actual increase because of the decline in the other nutrients after fermentation [31]. Similarly, decrease in the nitrogen-free extract and ether extract of GP could cause a relative increase in the ash content in this study.

Tannins can be divided into hydrolysable and condensed tannins [32]. Meini et al. [33] reported that *A. niger* produces tannase enzyme and degrades tannic acid (a hydrolysable tannin) in GP. However, condensed tannins are harder to be degraded than hydrolysable tannins because of the complicated structures [32]. This could be the reason for no change in the condensed tannin of GP after *A. niger* solid-state fermentation.

Solid-state fermentation increases radical scavenging activity by raising the amount and/or effectiveness of phenolic compounds in the fermentation medium [34]. However, *A. niger* can use some phenolic compounds as a carbon source during fermentation [35]. A reduction in the radical scavenging activity of GP can be due to *A. niger* consuming phenolic compounds.

### 4.2. Growth Performance

Viveros et al. [12] reported that dietary inclusion of 60 g/kg grape concentrate improved the FCR in broiler chickens. However, no changes in BW, FCR, and FI were reported in broiler chicks with dietary supplementation of 15 g/kg GP [3,36,37]. Similar findings were observed in broiler chickens fed diets containing GP at 10 [38], 20 [39], 30 [3], 60 [14,37,40], and 100 g/kg [13,41]. Nevertheless, Kumanda et al. [42] demonstrated that the FCR was impaired by dietary inclusion of GP at 55 and 75 g/kg, although 45 g/kg dietary GP did not affect the FCR. Similarly, a worsened FCR was reported in chicks receiving diets supplemented with 100 g/kg FP [43].

Oxidative stress, which is one of the important stressors in poultry production, can deteriorate the growth performance of broiler chickens [1]. Antioxidative compounds can improve broiler performance by eliminating the negative effects of oxidation [37]. In this study, dietary AO supplementation increased the BW of chickens. Similarly, Brenes et al. [37] indicated that dietary inclusion of vitamin E raised the antioxidant activity in serum and also enhanced the FCR in broilers. In this study, FGP increased the serum CAT level and raised the BW of chickens compared with the CON group. Niu et al. [44] reported an increase in serum GPx level and liver SOD and GPx level and an improvement in BW and FCR of broilers fed diets containing fermented *Ginkgo biloba* leaves. Furthermore, fermented *G. biloba* leaves increased the SOD level and total antioxidant activity of breast and thigh meat and also increased the body weight gain (BWG) and FCR in broiler chickens [25,45]. In contrast, Wu et al. [17] demonstrated that fermented pine needle did not alter the growth performance of chickens, although there was an increase in the GPx and SOD levels of serum and liver.

Grape pomace increased GPx and SOD in serum but did not change the growth performance of broiler chickens. Similarly, Ebrahimzadeh et al. [13] stated that 50 and 75 mg/kg dietary GP inclusion did not affect the BWG and FCR of broiler chickens, although GPx and SOD in plasma were increased. Brenes et al. [37] reported no change in BWG and FCR with 30 g/kg dietary GP, whereas there was increased serum antioxidant activity in broilers. Similarly, Goni et al. [3] reported that dietary GP did not alter the BWG and FCR of broiler chickens despite the antioxidant effect of GP observed with a decrease in MDA level of thigh meat.

The microbial population in the intestines plays important roles in digestion, absorption of nutrients, and control of pathogens and therefore, it has a huge impact on the growth performance of broiler chickens [46]. Improvement in the BW of chicks fed with FGP may be due to the decline in the *C. perfringens* count in the cecum in this study. Similarly, Zhang et al. [19] demonstrated that *G. biloba* leaves fermented with *A. niger* raised ileal *Lactobacillus* spp. and reduced cecal *Salmonella* spp. count and also enhanced the FCR in broilers. Moreover, dietary inclusion of fermented sour cherry kernel improved the FCR [47] and increased the cecal *Lactobacillus* spp. count [16] in broiler chickens.

Intestinal morphology, indicating the development and absorption capacity of the intestines, affects the growth performance of broiler chickens [48]. Viveros et al. [12] reported that 60 g/kg dietary grape concentrate improved the FCR of broilers by increasing VH:CD and LMT, and decreasing CD in the jejunum. Similarly, dietary grape proanthocyanidin increased the VH:CD and decreased the CD in the jejunum, and thereby improved the FCR in broilers at 7.5 and 15 mg/kg supplementation levels [49]. In the present study, dietary GP did not affect the VH, CD, and VH:CD in the ileum of broilers, although LMT was increased, which may be the reason for the lack of change in growth performance.

Meeting protein and amino acid requirements is important for taking the desired yield from broiler chickens. Cao et al. [25] showed that *A. niger* increased amino acids as well as crude protein content. An increase in crude protein and possible enhancement of amino acid composition may be another reason for the improvement in the BW of chicks fed with FGP. Similarly, *A. niger* increased crude protein content of sour cherry kernel with solid-state fermentation, and improved FCR was reported by dietary inclusion of fermented sour cherry kernel [47].

### 4.3. Carcass Characteristics, Meat and Liver Quality

Trampel et al. [50] noted that the L* value of the liver was increased by raising the fat level of the liver in broiler chicks. In this study, chicks fed diets containing AO had a higher L* value in the liver at 1 and 11 d of storage compared with the other treatment groups. This may be due to a possible increase in the fat level of the liver, although the abdominal fat was not changed among the treatment groups. On the other hand, lower pH in the liver of chicks from the AO group may have caused the higher L* value of the liver. Thus, Young et al. [51] showed that meat with lower pH was lighter than meat with higher pH.

Young et al. [51] indicated that a higher pH value can be correlated with strong antioxidant capacity. However, a reduction in the pH value of the liver was observed with dietary AO supplementation after 11 d of storage in the present study. This may be due to the insufficient antioxidative effect of dietary AO on broiler chicks because dietary supplementation of AO did not alter the serum GPx, SOD, and CAT levels and the MDA level of breast meat in this study. Furthermore, an increase in the b* value of the liver can be attributed to the lower pH level. Indeed, Qiao et al. [52] reported a negative correlation between the pH and b* values of breast meat in broiler chickens.

### 4.4. Antioxidant Activity

The activities of GPx, SOD, and CAT constitute the first-line antioxidant defense system and play an important role in protecting cells and tissues from the harmful effects of free-radicals. Therefore, higher levels of these enzymes in blood or tissues indicate a strong antioxidant capacity [53]. Grape pomace has strong antioxidant activity due to its phenolic and flavonoid compounds such as catechin, epicatechin, and procyanidin [37]. The GPx and SOD in serum were increased in chicks fed diets containing GP in this study. Similarly, dietary inclusion of 50 g/kg GP raised the plasma GPx and SOD level in broilers [13]. The total antioxidant activity in serum and plasma was increased by dietary inclusion of GP at 30 g/kg [37] and 100 g/kg [54]. Hosseini-Vashan et al. [14] reported a linear increase in plasma GPx and SOD levels in heat-stressed broilers fed diets containing 20, 40, and 60 g/kg GP. Similar to the results of the present study, 90 g/kg dietary GP increased the plasma-reduced glutathione and total antioxidant capacity but did not affect the CAT level of broiler chickens [7]. However, no change in total antioxidant capacity in the serum of broilers was observed by dietary supplementation of GP at 15 g/kg [3,37] and 20 g/kg [39]. Similarly, dietary GP did not affect reduced glutathione and oxidized glutathione in the serum and plasma of broiler chicks at the inclusion levels of 15 g/kg [36] and 100 g/kg [54].

Wu et al. [17] observed that *A. niger-*fermented pine needles increased the serum GPx and SOD levels in broiler chickens. In this study, FGP elevated the serum CAT level but did not change the GPx and SOD in the serum of broilers. Niu et al. [44] reported that the serum GPx level was increased by fermented *G. biloba* leaves, though the SOD and CAT levels were not altered. On the other hand, although antioxidant enzymes in serum were not changed by AO supplementation, GP increased GPx and SOD in serum and FGP raised the serum CAT level in this study. Similarly, Brenes et al. [37] demonstrated that total antioxidant activity in serum was elevated by 30 g/kg dietary GP but was not changed by dietary vitamin E supplementation.

### 4.5. Intestinal Bacterial Species

Grape pomace has antibacterial effects against various pathogenic bacteria thanks to its phenolic compounds [11]. It was reported that *C. perfringens* count in the ileum was reduced by supplementation of 50 g/kg GP [54] and 60 g/kg GP concentrate [12] in broiler diets. In this study, dietary GP did not affect the cecal *C. perfringens* count. Similarly, *C. perfringens* count in the cecum of broilers was not changed by dietary inclusion of 5 g/kg grape extract [48]. On the other hand, FGP decreased the cecal *C. perfringens* count of chickens in this study. Gogol et al. [55] demonstrated that *C. perfringens* count in the ileum was decreased by dietary supplementation of *A. niger* spores as a probiotic. Similarly, *A. niger*-fermented products also have a controlling effect on pathogen bacteria such as *E. coli* and *Salmonella* spp. in the intestines of broiler chickens [19,46]. The decline in cecal *C. perfringens* by dietary FGP may be caused by the probiotic effect of *A. niger*, secondary metabolites of fungi produced during fermentation, or unknown beneficial effects of the fermentation process.

An amount of 10 g/kg dietary grape seed reduced the *E. coli* and *Streptococcus* spp. count in the ileum of broiler chicks [56]. Similarly, Chamorro et al. [48] noted that dietary grape extract decreased the ileal *E. coli* counts in broiler chickens at the inclusion level of 5 g/kg. In the present study, dietary treatments did not affect the *E. coli* count in the cecum. Similarly, cecal *E. coli* count was not altered by dietary GP at the supplementation levels of 15 g/kg [36], 50 g/kg [54], and 60 g/kg [12]. Nevertheless, an increase in cecal *E. coli* count was reported for chicks fed diets containing 60 g/kg GP [12].

The coliform bacteria count in the ileum of broilers was decreased by dietary inclusion of fermented rapeseed meal [57] and fermented cottonseed meal [46]. Zhao et al. [58] indicated that fermented *G. biloba* leaves reduced the cecal *E. coli* count in laying hens. Dietary FGP did not affect the *E. coli* count in the cecum of chicks in this study. Similarly, no change in *E. coli* count was observed in the cecum of the broiler chickens fed diets containing fermented sweet cherry kernel [59], fermented sour cherry kernel [16], and fermented *G. biloba* leaves [19].

Lichovnikova et al. [36] demonstrated that dietary inclusion of GP increased the *Lactobacillus* spp. count in the ileum of broiler chickens. Similarly, supplementation of 60 g/kg GP concentrate in broiler diets increased the cecal *Lactobacillus* spp. and *Enterococcus* spp. counts [12]. Abu Hafsa and Ibrahim [56] reported an increase in ileal *Lactobacillus* spp. count with 10 g/kg dietary grape seed supplementation. In the present study, GP did not affect the cecal counts of *Lactobacillus* spp. and *Enterococcus* spp. Similar to the findings, no altered *Lactobacillus* spp. and *Enterococcus* spp. counts were reported in the ileum of broilers receiving diets supplemented with 50 g/kg GP concentrate [54]. However, dietary inclusion of 60 g/kg GP concentrate [12] and 5 g/kg grape extract [48] reduced the *Lactobacillus* spp. count in the ileum of broiler chickens.

Increased *Lactobacillus* spp. count was reported in the cecum of broilers receiving diets supplemented with the fermented sour cherry kernel [16]. Similarly, Zhang et al. [19] noted that inclusion of fermented *G. biloba* leaves in broiler diets raised the ileal *Lactobacillus* spp. count. Furthermore, *Lactobacillus* spp. count in the crop of broilers was elevated by dietary addition of sweet cherry kernel [59] and *G. biloba* leaves [19]. However, FGP did not change the cecal *Lactobacillus* spp. count in broilers. Similarly, no alteration was reported in cecal *Lactobacillus* spp. count of broilers receiving diets containing sweet cherry kernel [59] and *G. biloba* leaves [19].

### 4.6. Intestinal Morphology

A healthy intestinal morphology indicates better growth performance, effective nutrient absorption, and strong defense against pathogenic bacteria. A longer villus, shallower crypt, and thicker lamina muscularis are desired for healthy intestines [60,61]. Brenes et al. [37] reported that GP can protect intestines from possible oxidative damage during digestion due to its antioxidative polyphenolic compounds. Dietary inclusion of 60 g/kg GP concentrate increased the LMT and VH:CD, and decreased CD in the jejunum of broiler chickens [12]. In the present study, GP did not affect the intestinal morphology of the ileum compared with the CON group. Similar to the findings of the study, no change in VH and CD was reported in broilers fed diets supplemented with 5 g/kg grape extract [48]. However, Ebrahimzadeh et al. [13] showed that 50 g/kg dietary GP decreased the LMT in the ileum of the broiler chickens, although there was no change in the VH, CD, and VH:CD.

Zhang et al. [19] demonstrated that antioxidative compounds improve the intestinal morphology of chickens by supporting the antioxidant defense systems. Dietary AO supplementation elevated VH and VH:CD of the ileum in this study, even if no alteration occurred in the serum GPx, SOD, and CAT levels. However, Hosseini-Vashan et al. [14] noted that dietary GP did not affect the jejunal VH, CD, and VH:CD in chickens whose plasma GPx and SOD levels were increased by diets containing GP at 20, 40, and 60 g/kg levels. On the other hand, Ebrahimzadeh et al. [13] reported a decrease in VH, VH:CD, and LMT of the duodenum despite the increase in the plasma GPx and SOD levels of broiler chickens by 75 g/kg dietary GP.

Jazi et al. [46] reported a strong relationship between the intestinal morphology and microbial population. Viveros et al. [12] demonstrated that 60 g/kg dietary GP increased the VH:CD and LMT and decreased CD in the jejunum of broiler chickens whose cecal counts of *Lactobacillus* spp. and *Enterococcus* spp. were increased and *C. perfringens* count was decreased. However, dietary supplementation of FGP did not affect the intestinal morphology in the present study, although cecal *C. perfringens* count was lowered. On the other hand, dietary inclusion of FGP reduced the ileal LMT compared with the GP-supplemented group in the broilers of the present study. Considering that antioxidant compounds improve intestinal morphology [19], this result may be attributed to the decline in the radical scavenging activity of GP by the fermentation process.

## 5. Conclusions

This study showed that FGP has high potential to be an alternative to synthetic antioxidants by improving growth performance, supporting antioxidant capacity, and also by reducing the cecal *C. perfringens* count of broilers. Additionally, GP can be used in broiler diets with positive effects on the antioxidant defense system without detrimental effects on growth performance, although it caused lower growth performance than synthetic antioxidants. There is a need for further studies to determine the proper level of dietary FGP in broiler chickens.

## Figures and Tables

**Table 1:** Ingredients and nutritive values of basal diets.

Ingredients, g/kg	Starter (Days 1–11)	Grower 1 (Days 12–21)	Grower 2 (Days 22–35)	Finisher (Days 36–42)
Corn	197.0	244.0	217.0	257.5
Maize germ meal (9%)	230.0	230.0	230.0	230.0
Soybean meal (45%)	339.0	149.0	143.0	103.0
Full-fat soybean (35%)	100.0	125.0	80.0	80.0
Red dog (16%)	90.0	90.0	90.0	90.0
Maize germ (16%)	-	-	75.0	75.0
Sunflower meal (36%)	-	60.0	65.0	65.0
Chicken viscera (55%)	-	50.0	50.0	50.0
Meat and bone meal (35%)	-	25.0	25.0	25.0
Monocalcium phosphate (22.7% Ca)	12.4	3.9	1.5	1.5
Marble dust (36% Ca)	14.9	6.4	6.3	6.3
Salt	2.6	1.9	2.4	2.4
Liquid-Methionine (88%)	4.0	3.0	2.8	2.8
L-Lysine sulphate (55%)	4.6	6.8	7.0	7.0
L-Threonine (98%)	1.2	1.0	1.0	1.0
Vitamin and mineral premix ^1^	2.5	2.5	2.5	2.5
Sodium sulphate	1.2	1.0	1.0	1.0
Anticoccidial	0.6	0.5	0.5	-
Analyzed composition, g/kg, as fed				
Crude protein	243.0	228.0	213.7	190.7
Ether extract	56.9	59.2	72.9	70.2
Crude fiber	62.5	61.9	73.3	68.3
Ash	33.3	32.9	48.1	41.2
Calculated composition, g/kg, as fed				
Metabolic energy, MJ/kg	12.56	12.90	13.15	13.27
Lysine	15.8	14.8	14.3	13.3
Methionine	6.7	5.9	5.8	5.6
Methionine and cystine	11.0	10.3	10.3	9.9
Threonine	10.2	9.2	9.2	8.6
Tryptophan	3.0	2.6	2.5	2.3
Ca	9.6	11.2	10.8	10.6
Total P	7.4	8.3	8.0	7.9
Available P	4.9	5.7	5.1	5.1
Na	2.5	2.7	3.0	3.0

^1^ provided the following nutrients per kilogram of diet: 12,000 IU retinol; 2400 IU cholecalciferol; 40 mg α-tocopherol; 4 mg menadione; 3 mg thiamine; 6 mg riboflavin; 25 mg nicotinic acid; 10 mg pantothenic acid; 5 mg pyridoxine; 0.03 mg cyanocobalamin; 0.05 mg biotin; 1 mg folic acid; 80 mg Mn; 60 mg Zn; 60 mg Fe; 5 mg Cu; 0.2 mg Co; 1 mg I; 0.15 mg Se; 200 mg choline chloride.

**Table 2:** Effect of solid-state fermentation on the nutritional composition and radical scavenging activity of grape pomace ^1^.

Item (%, Dry Matter Basis)	GP	FGP	SEM	*p*-Value
Crude protein	12.6	28.3	3.51	<0.001
Ether extract	5.9	3.8	0.55	0.029
Ash	4.1	8.5	1.00	<0.001
Nitrogen-free extract	58.6	37.0	4.80	<0.001
Crude fiber	18.8	22.2	0.75	<0.001
Condensed tannin	10.4	12.8	0.55	0.641
Radical scavenging activity (DPPH)	94.3	68.6	6.14	0.006

Abbreviations: GP, grape pomace; FGP, fermented grape pomace; SEM, pooled standard error of mean. ^1^ Each value represents the mean of 3 replicate values.

**Table 3:** Growth performance of broiler chickens fed diets containing raw grape pomace, fermented grape pomace, and synthetic antioxidants ^1^.

Item	Day	CON	AO	GP	FGP	SEM	*p*-Value
BW, g	0	38.86	38.92	38.84	38.44	0.151	0.696
	21	983	1020	1020	1020	8.3	0.292
	42	3131 ^c^	3298 ^a^	3178 ^bc^	3229 ^ab^	18.2	0.003
FI, g	1–21	1197	1231	1247	1237	10.6	0.398
	21–42	3764	3782	3718	3698	23.7	0.597
	1–42	5002	5029	4940	4920	30.1	0.571
FCR, g:g	1–21	1.27	1.25	1.27	1.26	0.007	0.808
	21–42	1.72	1.68	1.75	1.70	0.009	0.053
	1–42	1.58 ^ab^	1.55 ^b^	1.60 ^a^	1.57 ^ab^	0.006	0.045

Abbreviations: BW, body weight; FI, feed intake; FCR, feed conversion ratio; CON, basal diet; AO, basal diet with 0.25 g/kg synthetic antioxidants (5% BHT, 1% BHA, and 11% EQ); GP, basal diet with 15 g/kg grape pomace; FGP, basal diet with 15 g/kg fermented grape pomace; SEM, pooled standard error of mean. ^a,b,c^ Means that have no superscript in common are significantly different from each other (*p* < 0.05). ^1^ Each value represents the mean of 5 replicate values (7 chickens per replicate).

**Table 4:** Liver quality parameters of broiler chickens fed diets containing raw grape pomace, fermented grape pomace, and synthetic antioxidants ^1^.

Item	CON	AO	GP	FGP	SEM	*p*-Value
Day 1						
pH	6.33	6.26	6.36	6.36	0.020	0.249
L*	30.76 ^b^	33.03 ^a^	30.69 ^b^	31.46 ^ab^	0.336	0.032
a*	16.12	16.48	16.11	16.90	0.233	0.618
b*	6.88	8.22	6.83	7.59	0.258	0.181
Day 5						
pH	6.15	6.08	6.19	6.18	0.018	0.109
L*	31.20	33.28	30.86	31.33	0.377	0.084
a*	16.21	15.48	15.55	15.94	0.264	0.773
b*	7.37	8.55	7.19	7.48	0.290	0.365
Day 11						
pH	6.15 ^a^	6.01 ^b^	6.16 ^a^	6.19 ^a^	0.024	0.030
L*	32.07 ^b^	35.76 ^a^	31.95 ^b^	32.10 ^b^	0.578	0.034
a*	15.28	15.59	14.22	14.14	0.340	0.337
b*	6.93 ^b^	8.95 ^a^	7.12 ^b^	6.62 ^b^	0.307	0.017

Abbreviations: L*, lightness; a*, redness; b*, yellowness; CON, basal diet; AO, basal diet with 0.25 g/kg synthetic antioxidants (5% BHT, 1% BHA, and 11% EQ); GP, basal diet with 15 g/kg grape pomace; FGP, basal diet with 15 g/kg fermented grape pomace; SEM, pooled standard error of mean. ^a,b^ Means that have no superscript in common are significantly different from each other (*p* < 0.05). ^1^ Each value represents the mean of 5 replicate values (1 chicken per replicate, 3 measurements per sample).

**Table 5:** Serum glutathione peroxidase, superoxide dismutase, and catalase of broiler chickens fed diets containing raw grape pomace, fermented grape pomace, and synthetic antioxidants ^1^.

Item, U/mL	CON	AO	GP	FGP	SEM	*p*-Value
GPx	256.0 ^b^	261.6 ^b^	368.2 ^a^	297.6 ^ab^	16.36	0.031
SOD	328.9 ^b^	314.1 ^b^	376.5 ^a^	302.4 ^b^	9.70	0.021
CAT	144.4 ^b^	134.1 ^b^	141.8 ^b^	169.8 ^a^	4.77	0.032

Abbreviations: GPx, glutathione peroxidase; SOD, superoxide dismutase; CAT, catalase; CON, basal diet; AO, basal diet with 0.25 g/kg synthetic antioxidants (5% BHT, 1% BHA, and 11% EQ); GP, basal diet with 15 g/kg grape pomace; FGP, basal diet with 15 g/kg fermented grape pomace; SEM, pooled standard error of mean. ^a,b^ Means that have no superscript in common are significantly different from each other (*p* < 0.05). ^1^ Each value represents the mean of 5 replicate values (1 chicken per replicate).

**Table 6:** Selected cecal bacterial species of broiler chickens fed diets containing raw grape pomace, fermented grape pomace, and synthetic antioxidants ^1^.

Item, log_10_ CFU g^−1^	CON	AO	GP	FGP	SEM	*p*-Value
*Lactobacillus* spp.	9.27	9.09	8.82	8.85	0.160	0.759
*Enterococcus* spp.	7.93	8.04	7.96	8.17	0.084	0.782
*Escherichia coli*	8.97	8.51	8.85	8.86	0.134	0.680
*Campylobacter jejuni*	6.12	6.23	6.20	6.42	0.073	0.552
*Staphylococcus aureus*	6.09	5.63	6.16	5.98	0.157	0.619
*Clostridium perfringens*	7.05 ^a^	7.02 ^a^	7.34 ^a^	5.95 ^b^	0.191	0.033

Abbreviations: CON, basal diet; AO, basal diet with 0.25 g/kg synthetic antioxidants (5% BHT, 1% BHA, and 11% EQ); GP, basal diet with 15 g/kg grape pomace; FGP, basal diet with 15 g/kg fermented grape pomace; SEM, pooled standard error of mean. ^a,b^ Means that have no superscript in common are significantly different from each other (*p* < 0.05). ^1^ Each value represents the mean of 5 replicate values (1 chicken per replicate).

**Table 7:** Intestinal morphology of broiler chickens fed diets containing raw grape pomace, fermented grape pomace, and synthetic antioxidants ^1^.

Item	CON	AO	GP	FGP	SEM	*p*-Value
Villus height, μm	687.2 ^b^	951.2 ^a^	740.7 ^b^	724.9 ^b^	21.55	<0.001
Crypt depth, μm	73.4	79.2	80.6	79.2	2.64	0.803
Villus height:crypt depth, μm:μm	9.4 ^b^	12.2 ^a^	9.4 ^b^	9.3 ^b^	0.43	0.041
Lamina muscularis thickness, μm	129.1 ^ab^	123.7 ^ab^	151.6 ^a^	102.8 ^b^	5.59	0.016

Abbreviations: CON, basal diet; AO, basal diet with 0.25 g/kg synthetic antioxidants (5% BHT, 1% BHA, and 11% EQ); GP, basal diet with 15 g/kg grape pomace; FGP, basal diet with 15 g/kg fermented grape pomace; SEM, pooled standard error of mean. ^a,b^ Means that have no superscript in common are significantly different from each other (*p* < 0.05). ^1^ Each value represents the mean of 5 replicate values (1 chicken per replicate).

## Data Availability

The data presented in this study are available on request from the corresponding author.
